# Effects of the Hedgehog Signaling Inhibitor Itraconazole on Developing Rat Ovaries

**DOI:** 10.1093/toxsci/kfab048

**Published:** 2021-04-27

**Authors:** Hanna Katarina Lilith Johansson, Camilla Taxvig, Gustav Peder Mohr Olsen, Terje Svingen

**Affiliations:** Division of Diet, Disease Prevention and Toxicology, National Food Institute, Technical University of Denmark, 2800 Kongens Lyngby, Denmark

**Keywords:** ovary, endocrine disruption, female reproduction, Ihh, Dhh, theca cells

## Abstract

Early ovary development is considered to be largely hormone independent; yet, there are associations between fetal exposure to endocrine disrupting chemicals and reproductive disorders in women. This can potentially be explained by perturbations to establishment of ovarian endocrine function rather than interference with an already established hormone system. In this study we explore if Hedgehog (HH) signaling, a central pathway for correct ovary development, can be disrupted by exposure to HH-disrupting chemicals, using the antifungal itraconazole as model compound. In the mouse Leydig cell line TM3, used as a proxy for ovarian theca cells, itraconazole exposure had a suppressing effect on genes downstream of HH signaling, such as *Gli1*. Exposing explanted rat ovaries (gestational day 22 or postnatal day 3) to 30 µM itraconazole for 72 h induced significant suppression of genes in the HH signaling pathway with altered *Ihh*, *Gli1*, *Ptch1*, and *Smo* expression similar to those previously observed in *Ihh/Dhh* knock-out mice. Exposing rat dams to 50 mg/kg bw/day in the perinatal period did not induce observable changes in the offspring’s ovaries. Overall, our results suggest that HH signal disruptors may affect ovary development with potential long-term consequences for female reproductive health. However, potent HH inhibitors would likely cause severe teratogenic effects at doses lower than those causing ovarian dysgenesis, so the concern with respect to reproductive disorder is for the presence of HH disruptors at low concentration in combination with other ovary or endocrine disrupting compounds.

Female reproductive health is dependent on appropriate ovary development, a process that initiates during fetal life. The ovaries arise from the same primordial structures as the testes, but are instructed to follow the female differentiation trajectory through a complex network of molecular signaling events (Nicol and Yao, 2014; Svingen and Koopman, 2013;). Development into fully functioning ovaries, from fetal life to reproductive age, involves coordinated events between oocytes, granulosa cells, theca cells, and other specialized cell types, with instructions from various signaling pathways such as wingless-like, fibroblast growth factors, retinoic acid, steroid hormones, and the Hedgehog (HH) pathway.

In recent years, there has been an increased focus on how early life exposure to environmental chemicals can potentially impact female reproductive health later in life (Buck Louis *et al.*, 2011; Johansson *et al.*, 2017). Traditionally, male reproductive health has been the main focus with respect to endocrine disruptors since male reproductive development is intrinsically linked to testicular androgen production. Female reproductive development, on the other hand, is not as dependent on steroid hormone signaling at early stages, hence the lack of research focus. But if not due to disrupted steroid signaling, what then could help explain the observed associations between fetal exposure to endocrine disruptors and reproductive disorders in women? One obvious explanation could be disrupted ovary development, which, in turn, would give rise to disease later as a consequence of compromised ovary/endocrine function (Johansson *et al.*, 2020).

In the ovary, HH signaling is important for reproductive development and function. Ablation of 2 of the 3 mammalian HH ligands, *Indian hedgehog* (*Ihh*) and *Desert hedgehog* (*Dhh*) leads to loss of theca cells, disrupted hormone homeostasis and infertility (Liu *et al.*, 2018). Also, disturbance to the downstream signaling pathway component *Smoothened* (*Smo*) leads to different reproductive problems, including longer estrous cycles and inhibited ovulation (Ren *et al.*, 2009). Establishment of the theca cell lineage around birth is dependent on HH signaling, and its disruption can compromise theca cell differentiation and function, ultimately affecting ovarian hormone production in adulthood (Liu *et al.*, 2015). The formation of the theca lineage occurs during a developmental time window considered sensitive to chemical perturbation; follicle assembly (Johansson *et al.*, 2017).

Although the mechanism of theca cell lineage specification is not completely understood, it appears that HH signaling is a key component. Granulosa cells express both IHH and DHH that likely act by paracrine action on surrounding mesenchymal stromal cells that express the *Patched 1* (*Ptch1*) receptor, thereby prompting cell differentiation (Nicol and Yao, 2014). This is very similar to how the fetal Leydig cells are recruited in the fetal testis by DHH expressed and secreted by Sertoli cells, then acting on surrounding PTCH1-positive mesenchymal cells (Barsoum *et al.*, 2009; Yao et al., 2002). By disrupting HH ligands in either ovaries or testes, differentiation of the steroidogenic cell lineages—theca and Leydig cells, respectively—is compromised. Thus, it can be speculated that, if a fetus is exposed to chemicals capable of disrupting HH signaling in the gonads, this could lead to disrupted ovary development and reproductive disorders similar to those manifesting if the sex hormone pathways are blocked (Johansson and Svingen, 2020).

There are several chemicals that can disrupt HH signaling, including drugs such as acetazolamide (Schreiner *et al.*, 2009) and itraconazole (Tiboni *et al.*, 2006), or environmental chemicals such as di(n-butyl) phthalate (Kim *et al.*, 2010b), piperonyl butoxide (Wang *et al.*, 2012), as well as maternal smoking (Fowler *et al.*, 2008). All these studies show that disrupted HH signaling can affect various organs and tissues across animal species—from fish to humans—with craniofacial and limb malformations being prevalent. To what extent the same chemicals can perturb gonadal development, however, remains largely unknown. Therefore, we selected itraconazole as a model compound to test whether such chemicals can impact ovary development in rats if exposure occurs during a sensitive developmental window; follicle assembly.

## MATERIALS AND METHODS

###  

####  

##### Cell culture

In the absence of an established theca cell line, we opted to use a testicular Leydig cell line and an ovarian granulosa cell line as proxy in this study to support *ex vivo* and *in vivo* findings in rat ovaries. The mouse immature Leydig TM3 cell line (Mather, 1980); ATCC CRL-1714^T^) and the granulosa KK-1 cell line (Kananen *et al.*, 1995)—gift in-kind from Dr Nafis Rahman, University of Turku, Finland—were cultured in Dulbecco's Modified Eagle's Medium (DMEM)/F-12 (1:1) (1×) Ham's F-12 nutrient mixture medium with 15 mM (4-(2-hydoxyethyl)-1-piperazineethanesulfonic acid (HEPES; Thermo Fisher Scientific), 50 mIU/ml penicillin, 0.5 mg/ml streptomycin (Life Technologies), and 10% fetal bovine serum (Life Technologies) at 37°C with 5% CO_2_ in a humidified atmosphere.

For RT-qPCR experiments, 1 ml of cell suspension/well was seeded in clear 12-well plates (Fisher Scientific). TM3 cells were seeded at a concentration of 4.0 × 10^5^ cells/ml and KK-1 cells at 2.0 × 10^5^ cells/ml. After 24 h, cells were treated with 100 nM smoothened agonist (SAG, cat. no. SML1314-1MG, Sigma Aldrich), to induce HH pathway components. This was done in both control and exposed groups. Simultaneously, cells in exposure groups were exposed to either 0.2 or 0.4 µM (TM3) or 0.2 or 5 µM (KK-1) of itraconazole (CAS 84625-61-6, cat.no. I6657, purity ≥ 98%, Sigma Aldrich). The concentrations of itraconazole were based on having no effect on cell viability. As the TM3 cells showed to be more sensitive than the KK-1 cells, the highest dose used was lower for the TM3 cells. Both SAG and itraconazole were dissolved in dimethyl sulfoxide (DMSO) and the same volume of DMSO was added to both control and exposed wells (<1%). Exposure continued for 48 h.

Cell viability was examined using the CellTiter-Glo 2.0 Cell Viability Assay (Promega). At experiment termination, 100 µl of cell viability assay reagent was added to each well. The plate was left on a shaker table for 10 min and here after luminescence was measured (EnSpire, PerkinElmer). Itraconazole exposure did not affect cell viability.

##### Rat ovary culture

Fetuses and pups from time-mated Sprague Dawley rats (Crl: CD [SD]; Charles River Laboratories, Sulzfeld, Germany) were used for *ex vivo* ovary culture. The dams were delivered on gestation day (GD) 10, with day of observed vaginal plug designated GD1. Ovaries were collected on GD22 and pup day (PD) 3 and placed on top of a filter approximately 6 mm in diameter (MFTM Membrane Filters, 0.45 µm HA, HAWP02500, Merck Millipore Ltd.) and floated on top of 400 µl of growth medium in 24-well plates (Greiner Bio-One International). The gonads were cultured at 37°C with 5% CO_2_ in a humidified atmosphere. Medium (containing itraconazole and/or DMSO) was changed at 24 and 48 h. The experiment was terminated after 72 h.

Itraconazole (CAS 84625-61-6, cat. no. I6657, purity ≥ 98%, Sigma Aldrich) was dissolved in DMSO (stock 10 mM). The medium used was DMEM/F-12 (1:1) (1×) F-12 nutrient mixture (HAM) medium with 15 mM HEPES (Thermo Fisher Scientific), 50 µg/ml Gentamicin (15710-049, 15710-080, Gibco by Life Technologies), 2.5 µg/ml Ampothericin (15290-026, Gibco by Life Technologies), and 10% fetal bovine serum (Life Technologies). The concentration of itraconzole in medium was 30 µM. This concentration allowed for a relatively high exposure without any viable toxic effects on the tissue. The same volume of DMSO was added to medium in the control group, corresponding to a DMSO concentration of < 1% in both groups.

**Figure 1.**
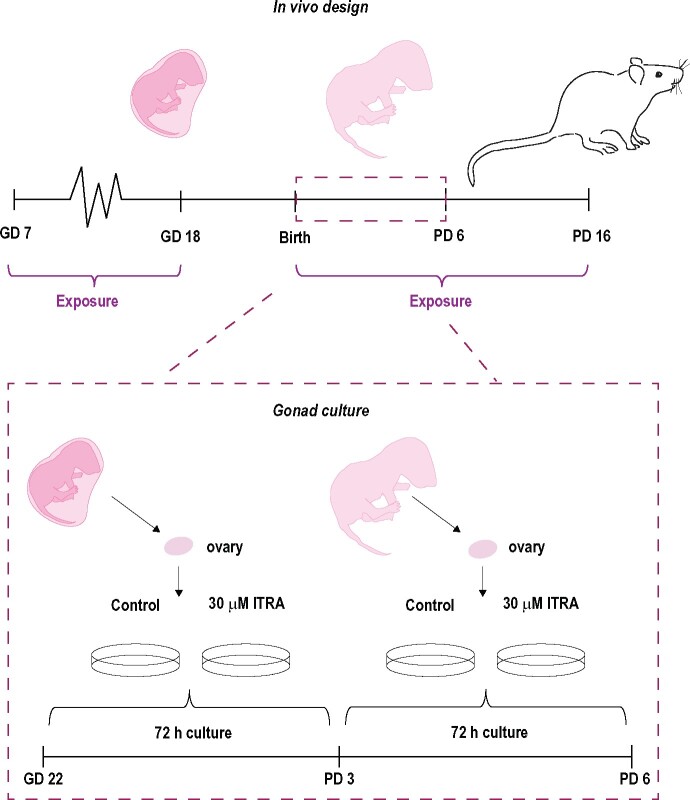
Design of the *in vivo* and *ex vivo* studies. In the *in vivo* study, rat dams were exposed to 50 mg/kg bw/day of itraconazole from gestation day (GD) 7 to 18 and again from the day after birth until pup day (PD) 16. Ovaries were excised and investigated on PD6 and PD16. In the *ex vivo* study, culture and exposure to 30 µM of itraconazole was conducted for 72 h in the early (GD22—PD3) and late (PD3–6) phase of follicle assembly. Ovaries were investigated on PD3 and PD6.

For assessment of early follicle assembly, ovaries were dissected directly into growth medium with the top part of the fallopian tubes (*infundibulum* and *fimbrae*) still attached on GD22 (the day before birth) and cultured for 72 h (until PD3). For investigation of the later phase of follicle assembly, ovaries were collected with the top part of the fallopian tubes (*infundibulum* and *fimbrae*) still attached on PD3 and cultured for 72 h (until PD6, Figure 1).

##### Animal study

Time-mated Sprague Dawley rats (Crl: CD [SD]) (Charles River Laboratories, Sulzfeld, Germany) were supplied on GD3. The day of vaginal plug was designated GD1 and the expected day of delivery (GD23) designated PD1. The animals were housed in pairs until GD18 and hereafter singularly in high temperature polysulfone (PSU) cages with Tapvei wooden shelters. The cages were placed in ScanTainers (Ventilated Cabinets from Scanbur) with controlled environmental conditions: 12-h light (21:00–9:00 h): 12-h dark (9:00–21:00 h) cycle, humidity 55% ± 5%, temperature 22°C ± 1°C and ventilation changing air 50–60 times/h. Animals were fed Altromin 1314 (soy and alfalfa free) and tap water (bisphenol A (BPA) free bottles 84-ACBT0702SU; PSU 700 ml w/ring square) *ad libitum*.

The day after arrival (GD4) the dams were distributed into 2 groups with similar weight (*n* = 5/group). They received vehicle (corn oil, C8267-2.5L, Sigma Aldrich) or 50 mg/kg bw itraconazole (CAS 84625-61-6, cat. no: J66390.06, batch no. P19F017, purity 98% [VWR]). The dose was chosen based on 2 previous rodent studies using oral administration of itraconazole during gestation. Tiboni *et al.* (2006) report signs of maternal toxicity at 150 mg/kg bw in mice and El-Shershaby *et al.* (2014) report teratogenicity at 100 mg/kg in rats. We therefore chose a dose of 50 mg/kg bw/day to be sure to avoid maternal toxicity and teratogenicity. Exposure was conducted once daily from GD7 to GD18, and then again from the day after birth until PD16. The break in exposure was introduced to allow for parturition as azole fungicides can induce labor complications (dystocia) in rodents (Noriega *et al.*, 2005; Taxvig *et al.*, 2007). Exposure was via gavage (2 ml/kg). A break in exposure from GD19 to the day after birth was due to previous studies with azole fungicides where we have observed problems with parturition. The animals were inspected for general toxicity twice per day. Ovaries were collected on PD6 and PD16 (Figure 1). The animal experiments were approved by the Danish Animal Experiments Inspectorate (Council for Animal Experimentation, license number 2015-15-0201-00553) and monitored by our in-house (DTU Food) Animal Welfare Committee.

##### Gene expression

Relative gene expression was assessed by RT-qPCR. To ensure sufficient RNA concentration, gonads from e*x vivo* cultures were pooled together in groups of 3, and PD6 *in vivo* ovaries in groups of 2 (1 ovary/animal from 2 siblings/litter). Each pool was considered 1 statistical unit. For PD16 ovaries, RNA was extracted from 1 ovary per litter. The procedure was undertaken as previously described (Svingen *et al.*, 2015). In short, total RNA was isolated using RNeasy Microkit (Qiagen) for gonads and RNeasy Minikit (Qiagen) for KK-1 and TM3 cells. RNA was quantified on a Nanodrop—1000 Spectrophotometer and cDNA synthesized from 500 ng RNA/sample (Omniscript, Qiagen) according to manufacturer’s description. RT-qPCR reactions were performed in duplicates on a QuantStudio 7 Flex Real-Time PCR System (Applied Biosystems) in 20 µl reactions containing TaqMan Fast Universal Master mix (Life Technologies), 3 µl diluted (1:20) cDNA and gene specific TaqMan assays (Life Technologies); *Ptch1* (Rn01527980_m1 [rat] and Mm00436029_m1 [mouse]), *Ihh* (Rn03810376_m1 [rat], and Mm00439613_m1 [mouse]), *Dhh* (Mm01310203_m1 [mouse]), *Gli1* (Rn01504237_m1 [rat] and Mm01160467_g1 [mouse]), *Smo* (Rn00563043_m1 [rat] and Mm01162710_m1 [mouse]), *Acta2* (Rn01759928_g1 [rat]), *Ddx4* (Rn01489814_m1 [rat]), *Foxl2* (designed in house, forward: ACG AGT GCT TCA TCA AGG TG, reverse: GGT AGT TGC CCT TCT CGA AC, probe: TAG TTG CCC TTG CGC TCG CC), amplification efficiency of 98% using a 6 serial 10-fold dilution in triplicates (rat). Data were analyzed by use of the comparative Ct method normalized with the geometric mean of 2 reference genes *Sdha* (Rn00590475_m1 [rat] and Mm01352363_m1 [mouse]) and *Rps18* (Rn01428913_gH [rat] and Mm02601777_g1 [mouse]). The suitability of these reference genes for the tissue in question was previously verified (Svingen *et al.*, 2015) and was monitored for cross-sample stability (Ct− values) in the present study.

##### Histology and immunofluorescence

Ovaries (*n* = 3/exposure group and age) were fixed in 10% formaldehyde and processed for mounting in paraffin. Tissue was sectioned at 5-µm thickness, then paraffin removed by heat treatment (60°C) for 30 min, washed in petroleum and hereafter rehydrated through a graded ethanol-water series.

For histological evaluation, slides were stained with hematoxylin and eosin, dehydrated through a graded water-ethanol series and mounted using Eukitt.

Immunofluorescence analyses were performed essentially as described previously (Svingen *et al.*, 2012). Briefly, antigen retrieval was achieved by 15 min heat treatment (microwave, 99°C) in Tris EDTA buffer (pH 9.0). The slides were then cooled at room temperature for 15–20 min, washed in PBS and blocked for 30 min at room temperature in 5% bovine serum albumin (BSA, cat. no. A6003, Sigma) in PBS. Primary-antibody for smooth muscle actin alpha (SMA-α, 1:800, cat. no. A2547, Sigma) was added in 1% BSA in PBS and incubated over night at 4°C. The following day, slides were washed in PBS and secondary antibody (AlexaFluor488 goat antimouse, cat. no. A32723, Thermo Fisher Scientific) was added in 1% BSA in PBS and left at room temperature for 1 h in the dark. Slides were washed with PBS and counterstained with 4',6-diamidino-2-phenylindole (DAPI; 1:1000, cat. no. 62248, Thermo Fisher Scientific) for 3 min. After washing with PBS and distilled water, slides were mounted with ProLong Gold Antifade Mountant (Thermo Fisher Scientific). Tissue sections were imaged with an Olympus BX-53 microscope and captured with a QImage Retiga-6000 monochrome camera and the Cell Sense Dimensions V1.16 software (Olympus Ltd., UK). Subsequent image processing was performed using Adobe Photoshop 2020 (Adobe Systems Inc., USA).

##### Statistical analysis

Statistical analysis was conducted in the software program GraphPad Prism 8.3.1 for Windows (GraphPad Software, San Diego, California, www.graphpad.com). A 2-tailed (unpaired) *t* test was used for normally distributed data. If data were not normally distributed or significant differences in variance was present, raw data were log transformed before analysis. If this did not improve normal distribution, nonparametric Mann Whitney test (with exact *p*-value) was used and if difference in variance was not improved 2-tailed Student’s *t* test with Welch’s correction was used.

## RESULTS

###  

#### HH Pathway Gene Expression in Itraconazole-Exposed Mouse TM3 and KK-1 Cells

**Figure 2.**
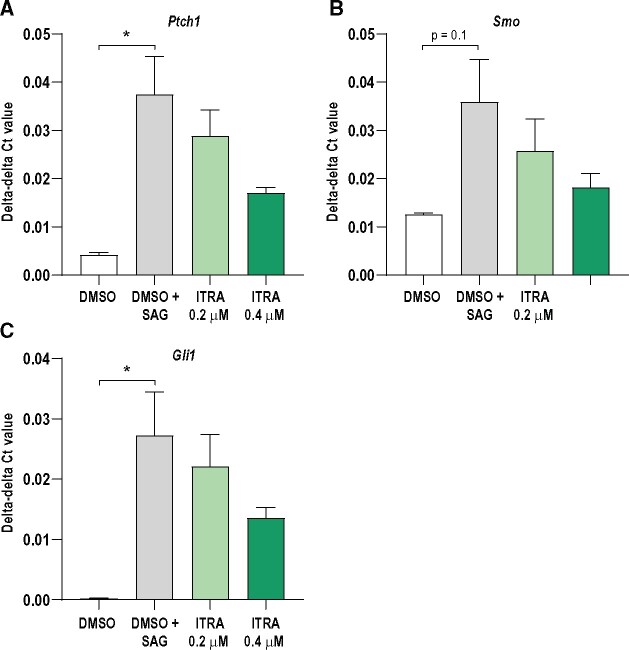
Expression of HH signaling components in TM3 cells exposed to DMSO only, DMSO + smoothened agonist (SAG) or DMSO + SAG + itraconazole (ITRA, 0.2 or 0.4 µM). In control samples, there is an upregulated gene expression in the presence of SAG compared with DMSO only. Effects of ITRA exposure was investigated by comparing DMSO + SAG control with DMSO + SAG + ITRA exposed cells (ANOVA). No significant effects were seen on expression of (A) *Ptch1*, (B) *Smo*, or (C) *Gli1* (*n* = 3/group, data shown as mean ± SEM, *p ≤ .05).

**Figure 3.**
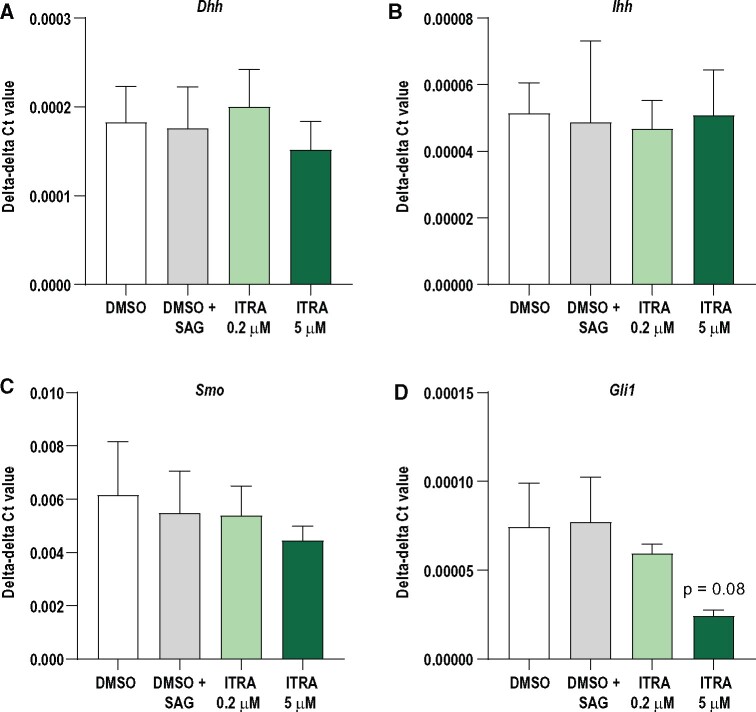
Expression of HH signaling components in KK-1 cells exposed to DMSO only, DMSO + smoothened agonist (SAG) or DMSO + SAG + itraconazole (ITRA, 0.2 or 5 µM). SAG did not affect the expression of HH signaling components as seen in the control groups (white and gray bars). Effects of ITRA exposure was investigated by comparing DMSO + SAG control with DMSO + SAG + ITRA exposed cells (ANOVA). No statistically significant effects were seen on expression of (A) *Dhh*, (B) *Ihh*, and (C) *Smo*. Expression of *Gli1* was downregulated, although not statistically significant (*p* = .08) (*n* = 3/group, data shown as mean ± SEM).

To test if itraconazole could disrupt HH signaling in cells relevant for endocrine cell lineages of the gonads, we performed exposure studies in 2 mouse cell lines. Since there is no established theca cell line, we used the mouse Leydig cell line TM3 as a proxy theca model for 2 reasons. First, Leydig cells are considered the male counterpart to theca cells, and secondly, we have previously shown that this cell line possesses active HH signaling components (Nygaard *et al.*, 2015). To further stimulate HH signaling *in vitro*, cells were also exposed to SAG; a well-established SMO agonists shown to for instance stimulate neuronal cell proliferation (Lewis and Krieg, 2014) and prostate cancer cell steroidogenesis (Lubik *et al.*, 2017). Stimulation with SAG upregulates the key HH downstream genes *Ptch1* (*p* = .02), *Smo* (*p* = .1), and *Gli1* (*p* = .04) in control cells (Figure 2, white and gray columns). Coexposure with itraconazole counteracts SAG-stimulated HH signal activation in a dose dependent manner, albeit not statistically significant (Figure 2). For the granulosa cell lineage, we used the mouse KK-1 cell line, which possesses the ability to synthesize steroid hormones such as progesterone and estradiol upon stimulation by human choriogonadotropin or follicle stimulating hormone (Kananen *et al.*, 1995). The basal expression of key HH signaling genes was lower than what was observed in TM3 cells and they did not respond to SAG stimulation (Figure 3). Thus, these KK1 cells are not very responsive to HH signal activation and only maintain a low basal activity. Exposure to itraconazle did reduce an already low expression level of *Gli1* as assessed by RT-qPCR, albeit not statistically significant (*p* = .08). The upstream factors *Ihh*, *Dhh*, and downstream factor *Smo* were not significantly changed in the exposed versus control cells, also reflecting the non-responsiveness of KK-1 cells to SAG stimulation (Figure 3).

Taken together, results from TM3 and KK-1 cell suggest the signal transduction downstream of SMO might be affected in endocrine cells of the mouse gonads. This is in agreement with previous characterization of itraconazole as a SMO inhibitor (Kim *et al.*, 2010a). We observed no cytotoxicity in neither TM3 nor KK-1 cells at the tested concentrations (data not shown).

#### HH Pathway Gene Expression Is Altered in *Ex Vivo* Rat Ovaries Exposed to High Dose of Itraconazole, But Not in *In Vivo* Rat Ovaries Exposed to Low Dose of Itraconazole

As itraconazole is a known HH signaling disruptor (Kim *et al.*, 2010a), and has some effect on steroidogenic cells of the gonads, we wanted to test whether perinatal exposure could disrupt these cells in intact ovaries. For this we chose to expose rat ovaries both *ex vivo* and *in vivo*. The rationale for selecting both systems was that the potential for general toxicity in pregnant rats at higher doses of itraconazole would prevent us from achieving internal doses approaching those of the *in vitro* experiments. Thus, the inclusion of explanted rat ovaries would allow for higher exposure doses that would aid in the characterization of molecular mechanisms of effect.

**Figure 4.**
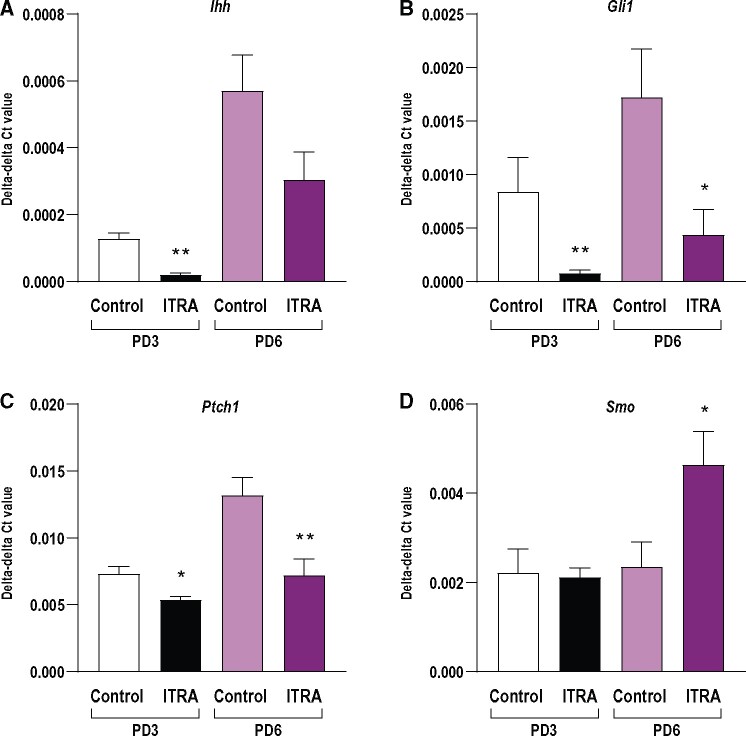
Expression of genes central to the HH signaling in *ex vivo* cultured ovaries (A) *Ihh* expression was significantly downregulated at pup day (PD) 3 (*p* = .004) after *ex vivo* exposure to itraconazole (ITRA). A similar tendency was seen in *ex vivo* exposed ovaries on PD6, but was not statistically significant (*p* = .09). B and C, The expression of *Gli1* and *Ptch1* was significantly downregulated at both PD3 and PD6 after *ex vivo* exposure. D, Expression of *Smo* was significantly upregulated on PD6 after *ex vivo* exposure (*p < .05, **p < .01, *n* = 3–5/group, data shown as mean ± SEM).

In the e*x vivo* experiments, we chose to expose the explanted ovaries to 30 µM itraconazole, which we estimated would be roughly 10–15 times higher than what would be achieved in the *in vivo* exposure regimen. In ovaries cultured in the presence of itraconazole from GD22 to PD3, *Ihh* expression was significantly downregulated (*p* = .004), indicating that less IHH, 1 of the HH ligands, may be produced at this age. Expression of *Ihh* showed a similar tendency in ovaries exposed from PD3 to PD6, but was not statistically significant (*p* = .09; Figure 4A). The expression of *Gli1* (*p* = .01, *p* = .02) and *Ptch1* (*p* = .02 and *p* = .01) were significantly downregulated after exposure to itraconazole in both the PD3 and PD6 ovaries, indicating a significant disruption of the HH signaling pathway (Figs. 4B and 4C) Expression of *Smo* was not affected at PD3 in cultured ovaries, whereas in the more mature group, on PD6, there was a significant increase in *Smo* expression after exposure (*p* = .04, Figure 4D). This latter effect could indicate at compensatory mechanism activated in response to itraconzole exposure.

**Figure 5.**
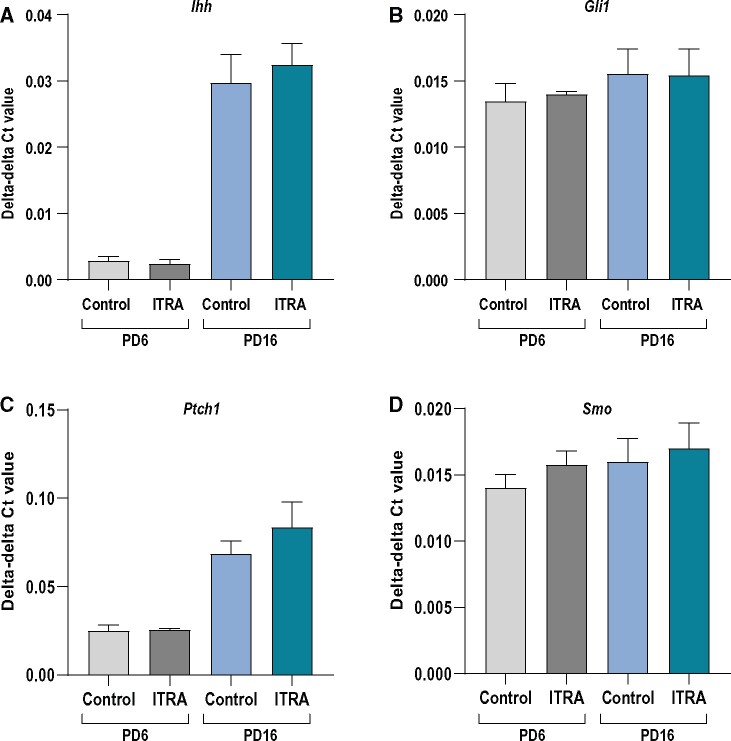
Expression of HH signalling components in ovaries from offspring exposed to 50 mg/kg bw/day itraconzole (ITRA) in the perinatal period (gestational day [GD] 7–18 and again from birth to pup day [PD] 16). There were no significant effects on the expression of (A) *Ihh*, (B) *Gli1*, (C) *Patch1*, or (D) *Smo* at PD6 nor PD16 (*n* = 4–5/group, data shown as mean ± SEM).

For the *in vivo* study, pregnant dams were exposed to 50 mg/kg bw/day from GD7 to GD18, with a break in exposure to allow for parturition (azole fungicides can cause dystocia in rodents; Noriega *et al.*, 2005; Taxvig *et al.*, 2007), and again during lactation from the day after parturition to PD16 when the study was terminated. Using this dose, we did not see any maternal toxicity or problems with parturition and bodyweights were not significantly affected in exposed offspring relative to control animals (data not shown). Gene expression of *Ihh*, *Gli1*, *Ptch1*, and *Smo* was not affected in ovaries of the offspring on PD6 or PD16 (Figs. 5A–D). Expression of the germ cell marker *Ddx4* and granulosa cell marker *Foxl2* was also unchanged (data not shown).

**Figure 6.**
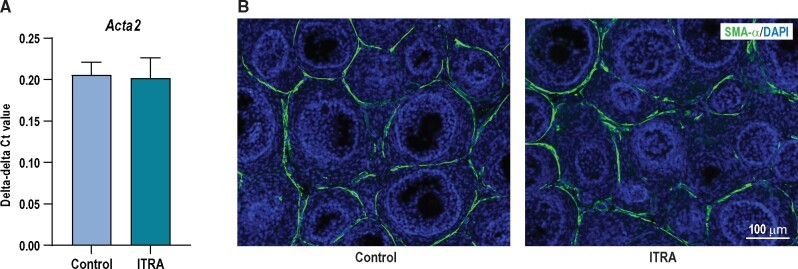
Expression of smooth muscle actin alpha (SMA-α) on pup day (PD) 16 was not affected by itraconazole (ITRA) exposure. (A) There was no effect on expression of *Acta2* at gene level (the gene coding for SMA-α protein), (B) and no difference in localization or protein expression (semiquantitative evaluation) could be identified between control group and ITRA exposed group (data shown as mean ± SEM, *n* = 4–5).

SMA-α has been shown to be sensitive to downregulation of the HH pathway (Liu *et al.*, 2018). As the theca layer containing the SMA-α is not fully established on PD6, one of the reasons for continuing the study until PD16 was to look at this marker. However, no effects were seen on either gene expression or on protein level on PD16 (Figure 6).

## DISCUSSION

In this study, we wanted to investigate if the HH signaling pathway in the ovary can be perturbed by chemical exposure and thereby affect female endocrine and reproductive function. For this purpose, we exposed gonadal cells, explanted ovaries, and pregnant rats to itraconazole; a pharmaceutical agent known to inhibit HH signaling (Kim *et al.*, 2010a). Itraconazole has been used as a medicinal antifungal agent for nearly 3 decades and is known for its broad activity spectrum and established efficacy and safety in human patients (Caputo, 2003). Thus, our intention was purely to use itraconazole as a model compound for HH inhibition in developing rodent ovaries.

HH signaling is central to ovary development and function as shown in a mouse double knock-out (KO) model where the combined ablation of *Dhh* and *Ihh* causes infertility by compromised folliculogenesis (Liu *et al.*, 2018). Interestingly, the expression profile for HH signaling in the *Ihh/Dhh* double KO ovaries and the single *Ihh* KO ovaries are comparable to what we observed in our ovary explant model with itraconazole exposure; a significant downregulation of *Ptch1* and *Gli1*. Downregulation of these genes can have severe implications for ovary function, as expression of *Gli1* is central for establishment of a functional theca cell layer. Already around the time of birth, the theca progenitor cells are prompted to commit to the theca cell lineage by acquiring *Gli1* expression. These *Gli1* positive cells represent a progenitor pool for the cells that will become theca cells in the adult when stimulated by IHH and DHH synthesized by granulosa cells (Liu *et al.*, 2015). We thus hypothesize that the downregulation of *Gli1* in the PD3 and PD6 *ex vivo* ovaries exposed to itraconazole compromises theca cell recruitment. This will lead to problems with follicular function, such as hormone production and fertility in the adult female rats, as previously shown in mice (Liu *et al.*, 2015, 2018). In further support of this hypothesis, is the observation that *Smo* was upregulated in the PD6 *ex vivo* ovaries. This could indicate a compensatory mechanism in response to reduced signaling downstream of SMO. Indeed, upregulated SMO signaling can interfere with establishment of the smooth muscle cells in the theca layer resulting in lack of capacity to ovulate (Ren *et al.*, 2009). Establishment of smooth muscle cells in the theca layer is also a problem in the *Ihh/Dhh* double KO ovaries where absence of the SMA-α is seen (Liu *et al.*, 2015, 2018). As the theca layer is identifiable approximately 1 week after birth in rodents (Edson *et al.*, 2009), we investigated the level of SMA-α in PD16 ovaries exposed to itraconazole *in vivo*. We identified a clear theca layer, but did not see an effect on SMA-α in exposed ovaries. This is not surprising as HH signaling components in these ovaries were not affected on either PD6 or PD16.

Previous *in vivo* rodent studies using oral administration of itraconazole during gestation report clinical signs of maternal toxicity at 150 mg/kg bw in mice (Tiboni *et al.*, 2006) and teratogeneicity at 100 mg/kg in rats (El-Shershaby *et al.*, 2014). Since both studies were terminated before birth, we could not evaluate if there would be problems with parturition. This is something we would suspect based on own observations from various azole fungicide exposure studies (data not shown). Hence, to minimize the risk of maternal toxicity, teratogenicity, and problems with parturition, we opted for a dose of 50 mg/kg bw/day, including a break from exposure between GD18 to the day after birth. With this dose, we did not observe any effects on ovarian HH signaling components, as was clearly observed *ex vivo* and with a tendency *in vitro*. A likely explanation for this is difference in itraconazole concentrations in the target cells/tissues, with the *in vivo* situation representing a much lower internal exposure. Based on a previous study with the azole fungicides flusilazole and triticonazole, where internal concentrations in fetal plasma after maternal oral exposure was measured (Draskau *et al.*, 2019), we would roughly estimate itraconazole levels in fetal blood to be around 2 µM, assuming similar metabolism of the azoles. If this rough estimate is correct, it would mean that the concentrations where we saw effect in the *ex vivo* organ culture was 15 times higher (30 µM) than *in vivo*. Notably, we also paused exposure from GD18 to the day after birth to circumvent potential complications with parturition. This is an important period in relation to theca cell commitment (Liu *et al.*, 2015), and the lack of effects seen on the theca cell layer, such as normal smooth muscle cells, may be due to this. Nevertheless, with the *ex vivo* ovaries showing clear disruption to the HH pathway, perinatal exposure to HH-disrupting chemicals can interfere with ovary development, depending on exposure level as well as timing of exposure. Importantly, disrupted HH signaling can cause severe teratogenic effects (Varjosalo and Taipale, 2008), and these effects may occur at exposure levels below those that would be required to affect ovary development. As shown herein, gestational exposure to 50 mg/kg bw/day itraconazole induced no severe toxicity or teratogenicity, nor any effects on the ovaries; but a dose of 100 mg/kg bw/day induced teratogenicity in a previous study (El-Shershaby *et al.*, 2014), which is why we opted not to expose our rats to these high doses as it would render any potential effects on ovary development of secondary interest. Reproductive-specific effects at lower doses of HH inhibitors would therefore more likely only manifest in combination with inhibition of other hormone-sensitive signaling pathways, as recently discussed (Johansson and Svingen, 2020).

Apart from itraconazole and other pharmaceuticals, several environmental chemicals have been shown to disrupt HH signaling. This includes the insecticide synergist piperonyl butoxide (Wang *et al.*, 2012), di(n-butyl) phthalate (Kim *et al.*, 2010b), tributyltin (Zhang et al., 2012), methoprene photolytic compounds (Smith *et al.*, 2003), and cigarette smoke (Fowler *et al.*, 2008). Common amongst these chemicals is that they can all antagonize HH signaling, albeit at various steps of the signaling cascade. This includes reduced HH ligand expression and blocking SMO activation. The concern is therefore that exposures to such chemicals at critical developmental life stages could compromise reproductive development and future function. Developmental disruption of HH signaling is known to cause many severe disorders, not least skeletal malformations (Varjosalo and Taipale, 2008), whereas its impact on reproductive parameters is less explored. As mentioned earlier, it can be easily argued that we should not be concerned about potential effects on reproductive health for HH signaling disruptors that causes severe teratogenic effects at exposure levels below those causing an effect on reproductive organs. The concern, however, is that exposure to lower doses of the same compounds can contribute to reproductive disorders by more subtle effects on, for instance, the ovaries during development. In combination with other endocrine disrupting events, the compromised offspring would be more susceptible for developing reproductive disease when exposed to additional ovary- or hormone-disrupting compounds.

In conclusion, we have shown that perinatal exposure to a chemical that has the capacity to antagonize the HH pathway may disrupt rodent ovary development. Although we saw no adverse effect *in vivo* in our study, it is likely that HH perturbation could affect theca cell recruitment as shown in vitro and similarly to effects postulated to take place in Leydig cells of the testes (Johansson and Svingen, 2020). This would ultimately affect folliculogenesis, with negative impacts on fertility later in life, but also other female reproductive disorders such as polycystic ovarian syndrome, premature ovarian insufficiency and ovarian cancers, as these diseases are linked to compromised steroid hormone signaling originating from the ovarian steroidogenic cells, not least the theca lineage. 

## DECLARATION OF CONFLICTING INTERESTS

The authors declared no potential conflicts of interest with respect to the research, authorship, and/or publication of this article. 
